# Relevance of Receptor for Advanced Glycation end Products (RAGE) in Murine Antibody-Mediated Autoimmune Diseases

**DOI:** 10.3390/ijms20133234

**Published:** 2019-07-01

**Authors:** Alexandra Eichhorst, Christoph Daniel, Rita Rzepka, Bettina Sehnert, Falk Nimmerjahn, Reinhard E. Voll, Nina Chevalier

**Affiliations:** 1 Department of Rheumatology and Clinical Immunology, Medical Centre—University of Freiburg, Faculty of Medicine, 79106 Freiburg, Baden-Wuerttemberg, Germany; 2 Department of Nephropathology, Friedrich-Alexander University (FAU) of Erlangen-Nuremberg, 91054 Erlangen, Bavaria, Germany; 3 Department of Biology, Friedrich-Alexander University (FAU) of Erlangen-Nuremberg, 91058 Erlangen, Bavaria, Germany

**Keywords:** RAGE, RA, arthritis, SLE, lupus, HMGB1

## Abstract

It is incompletely understood how self-antigens become targets of humoral immunity in antibody-mediated autoimmune diseases. In this context, alarmins are discussed as an important level of regulation. Alarmins are recognized by various receptors, such as receptor for advanced glycation end products (RAGE). As RAGE is upregulated under inflammatory conditions, strongly binds nucleic acids and mediates pro-inflammatory responses upon alarmin recognition, our aim was to examine its contribution to immune complex-mediated autoimmune diseases. This question was addressed employing RAGE−/− animals in murine models of pristane-induced lupus, collagen-induced, and serum-transfer arthritis. Autoantibodies were assessed by enzyme-linked immunosorbent assay, renal disease by quantification of proteinuria and histology, arthritis by scoring joint inflammation. The associated immune status was determined by flow cytometry. In both disease entities, we detected tendentiously decreased autoantibody levels in RAGE−/− mice, however no differences in clinical outcome. In accordance with autoantibody levels, a subgroup of the RAGE−/− animals showed a decrease in plasma cells, and germinal center B cells and an increase in follicular B cells. Based on our results, we suggest that RAGE deficiency alone does not significantly affect antibody-mediated autoimmunity. RAGE may rather exert its effects along with other receptors linking environmental factors to auto-reactive immune responses.

## 1. Introduction

Systemic lupus erythematosus (SLE) and rheumatoid arthritis (RA) are chronic inflammatory autoimmune diseases. Although the etiopathogenesis of these diseases is not completely understood, it is assumed they result out of the complex interplay between genetic and environmental factors [1,2]. The generally broad spectrum of clinical manifestations is caused by a break of tolerance that results in immune dysregulation and autoantibody production, followed by immune complex formation and activation of complement and inflammatory cascades [3,4].

In SLE and RA, the presence of autoantibodies has provided clues to the underlying disease pathogenesis. Generally, in both disease entities, several autoantibodies to diverse antigens can be detected. In RA, rheumatoid factor and anti-citrullinated protein antibodies are the most prominent, while recently, additional autoantibodies against post-translationally modified proteins were discovered, such as anti-acetylated or anti-carbamylated protein antibodies [5]. Even more in SLE, a broad spectrum of autoantibodies directed to diverse self-antigens has been reported. In particular, these comprise nuclear components, such as double-stranded DNA (dsDNA), RNPs or nucleosomes [6]. 

The pathogenesis of humoral autoimmunity has been the focus of many studies. Autoantibodies are produced by autoreactive B cells that have escaped central tolerance mechanisms and undergo clonal expansion when continuously exposed to antigen as well as triggering genetic and environmental factors. Here, it still remains a matter of debate how poorly immunogenic molecules and seemingly irrelevant self-antigens can become targets of humoral immunity in autoimmune diseases. Recent studies indicate that associations between pathogen-associated molecular patterns (PAMPs) or danger-associated molecular patterns (DAMPs) and development of autoimmunity constitute an important level of regulation. DAMPs and PAMPs are recognized by pattern recognition receptors (PRRs) on immune cells, such as monocytes or macrophages. This triggers intracellular signal transduction cascades and activates transcription factors, such as NF-κB, leading to the production of pro-inflammatory cytokines [7]. The resulting inflammation modifies the activation threshold that may be necessary for the break of peripheral tolerance and to the tip of balance towards initiation of autoimmunity [8]. 

One such PRR is the receptor for advanced glycation end products (RAGE). RAGE is a transmembrane receptor of the Ig superfamily and found to be expressed in a variety of tissues, such as epithelial cells of the lung, cells of the central nervous system, kidneys, heart and vasculature as well as immune cells—T and B cells, monocytes, dendritic cells (DCs), and granulocytes [9]. RAGE was first described as a binding partner for advanced glycation end products (AGEs) [10], it, however, interacts with further heterogeneous ligands, such as proteins from the S100 family, amyloid fibrils, high mobility group box 1 (HMGB1), C3a, and CpG DNA oligonucleotides [11,12,13]. By binding to its diverse ligands, RAGE activates signaling cascades, such as activation of mitogen -activated protein kinase, p21ras and NF-κB translocation inflicting the upregulation of inflammatory molecules [13,14,15,16,17].

Under normal physiologic conditions RAGE is expressed at low levels but highly upregulated under chronic inflammation as a result of the accumulation of various ligands [18,19,20,21,22]. It is therefore obvious that increased RAGE signaling has been associated with the development and progression of pathological states, such as Alzheimer’s disease, diabetes, cardiovascular disease, cancer and further inflammatory diseases [9,23,24,25].

RAGE also exists in a soluble form (sRAGE) which lacks the transmembrane and cytosolic domains of the full-length RAGE. sRAGE interacts with the same ligands and may act as ‘decoy’ receptor that binds ligands and prevents their interaction with full-length cell membrane-bound RAGE [26,27]. Consequently, increased sRAGE levels decrease full-length RAGE signaling and the resulting inflammation [21,28].

HMGB1 is a nuclear DNA-binding protein and a ligand with high affinity to RAGE. When localized intracellularly, HMGB1 participates in biological processes, such as the regulation of transcription by interacting with DNA chromatin structure. However, when released into the extracellular milieu upon secretion or release from primary or secondary necrotic cells, HMGB1 acts as DAMP and promotes an inflammatory immune response [29,30,31]. The fact that HMGB1 binds DNA with strong affinity and acts as a pro-inflammatory mediator when released from cells potentially makes it an ideal contributor to inflammation in immune complex-mediated diseases. Accordingly, HMGB1 was shown to be complexed to nucleosomes in secondary necrotic cells as well as in circulating immune complexes from SLE patients [32] and may be critical for the pro-inflammatory activity of lupus-associated immune complexes. Hence, by rendering mammalian DNA through complexation more immunogenic, extracellularly released HMGB1 may have potent immune-stimulatory properties and may importantly contribute to the loss of tolerance in autoimmunity. Furthermore, studies on the expression of one of HMGB1’s favorite receptors, RAGE, and sRAGE in autoimmune tissues and their biological effects support a possible contribution of the HMGB1—RAGE axis to autoimmune pathogenesis. Nevertheless, the exact role of RAGE in antibody-mediated autoimmunity, such as RA and SLE, is still unclear since results from earlier studies are in part contradictory. The aim of this study was, therefore, to clarify the relevance of RAGE-signaling in the development of antibody-mediated autoimmunity on the basis of murine models employing RAGE−/− animals.

## 2. Results

### 2.1. Influence of RAGE Deficiency in Pristane-Induced Lupus

Based on the hypothesis that RAGE may represent an important linker between environmental factors and autoreactive immune responses, we first aimed to examine whether RAGE deficiency influences the production of autoantibodies and development of lupus pathology. Apart from classic mouse models of spontaneous lupus, in which genetic factors play the major role, also exposure to environmental triggers, such as pristane (2, 6, 10, and 14 tetramethylpentadecane, TMPD), can induce lupus [33]. To that end we injected pristane into WT and RAGE−/− animals and determined development of serum anti-dsDNA antibody levels and nephritis five months (replicate 1: *n* = 6 RAGE−/− and 7 WT mice), six months (replicate 2: *n* = 6 RAGE−/− and 8 WT mice) and seven months (replicate 3: *n* = 15 RAGE−/− and 10 WT mice) later. Three different end points were chosen as according to our experience, clinical symptoms develop five to seven months after injection but show a relatively high degree of variability regarding the time point they first appear and their severity. 

**Figure 1:**
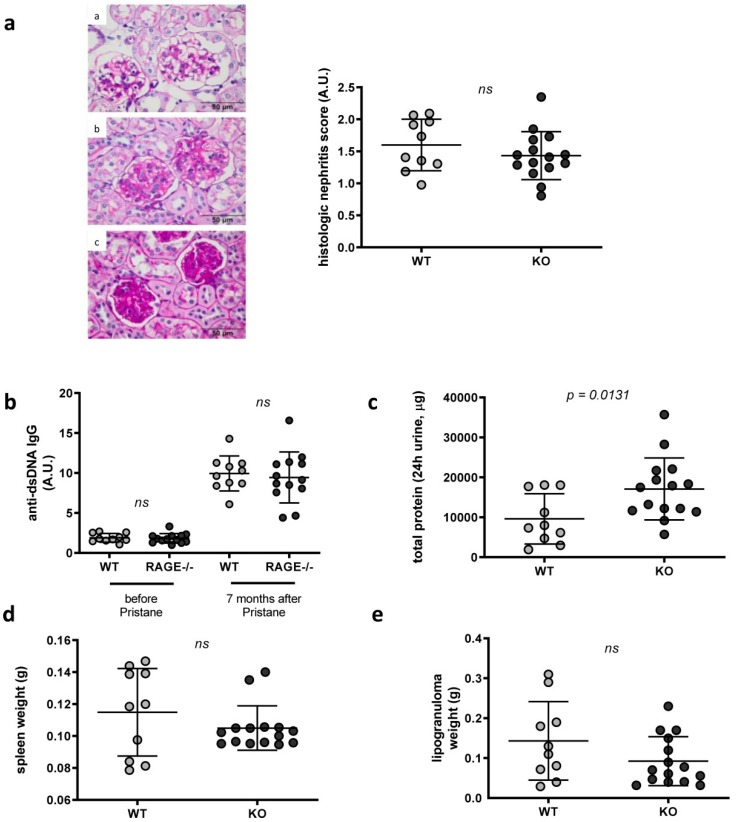
Influence of RAGE deficiency on disease development in pristane-induced lupus. C57BL/6 (WT) and RAGE−/− mice were injected intraperitoneally with 0.5 mL pristane. Serum, urine, spleen and kidney samples, as well as intraperitoneal lipogranulomas, were collected seven months later (replicate 3: *n* = 15 RAGE−/− and 10 WT mice). (**a**) Representative kidney sections stained with periodic acid Schiff stain (PAS). Depicted are glomeruli with grade I (**a**), II (**b**) and III (**c**) glomerulonephritis as well as cumulative nephritis scores of RAGE−/− compared to WT animals. (**b**) The concentrations of anti-dsDNA autoantibodies were determined by ELISA before and seven months after injection. Note, that only 13 of originally 15 RAGE−/− mice were examined in this experiment, as not enough material was left in two samples. Pristane injection induces a ca. five-fold increase of anti-dsDNA autoantibodies in both RAGE and WT animals. (**c**) Protein concentrations were determined using a BCA protein assay in urine samples collected for 24 hours using metabolic cages. (**d**,**e**) Weights of spleen and lipogranulomas. (**a**–**e**) Each data point represents a single mouse; values are indicated as mean ± SD. Unpaired Mann–Whitney *U*-test was used for statistical analysis.

Compared to NZB/W F1 animals, injection of pristane into C57BL/6 (WT) animals, only induced mild lupus-like symptoms and lower anti-dsDNA antibody levels (Supplement Figures S6 and S7a). Nevertheless, a significant increase in anti-dsDNA autoantibodies could be detected after pristane injection (ca. five-fold in RAGE−/− and WT animals seven months after pristane injection and ca. 2.3–3.7 fold in RAGE−/− and WT animals five months after pristane injection) (Figure 1b, Supplement Figure S1a). Histologic examination did not reveal any difference between both groups (Figure 1a, Supplement Figure S2a), while proteinuria—although generally very low (Supplement Figure S7)—was tendentiously increased in RAGE−/− animals (Figure 1c, Supplement Figure S1c,d). In contrast to that, in two of three independent experiments, anti-dsDNA antibody production was higher in WT than in RAGE−/− mice (Figure 1b, Supplement Figure S1a,b).

In view of the fact that the differences between the groups were only weak and mostly not fully consistent between the independently repeated experiments, we conclude that RAGE deficiency alone does not significantly affect lupus development. This is further supported by the fact that RAGE deficiency significantly changed lymphoproliferation as determined by spleen weight only in one of three independent experiments (Figure 1d, Supplement Figure S2b,c), peritoneal lipogranuloma formation was not different in RAGE−/− compared to WT animals (Figure 1e, Supplement Figure S2b,c).

### 2.2. Phenotype of Splenocytes in WT and RAGE−/− Mice after Pristane Injection

**Figure 2:**
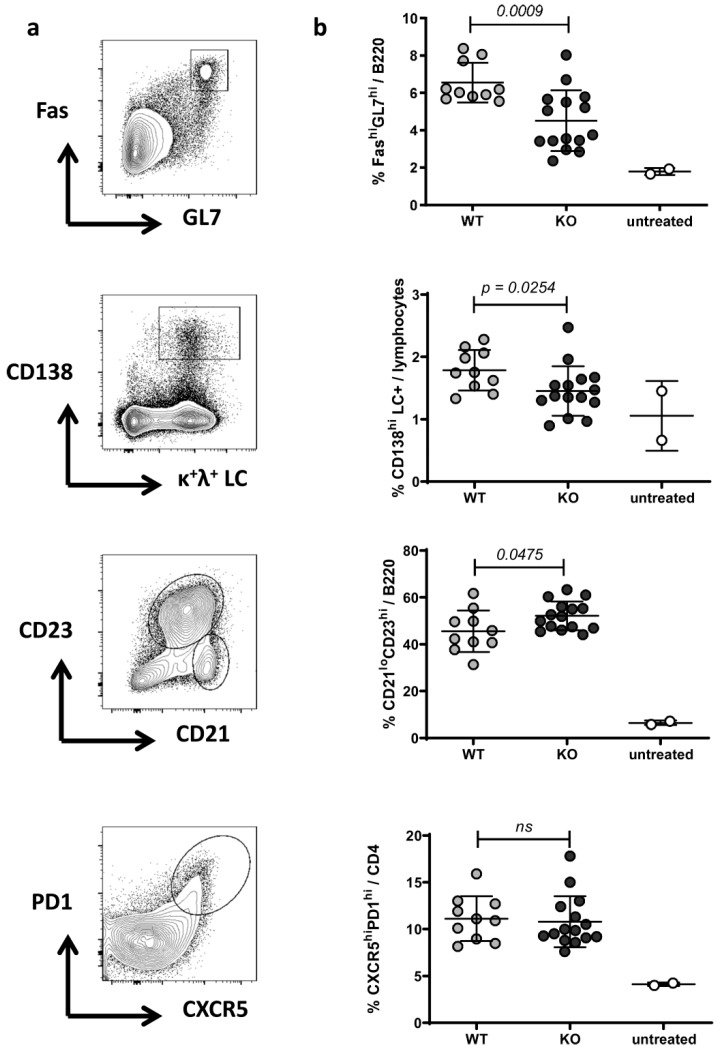
Phenotype of splenocytes in WT and RAGE−/− mice after pristane injection. The analysis was performed by flow cytometry seven months after pristane injection (replicate 3: *n* = 15 RAGE−/− and 10 WT mice). Depicted are (**a**) representative FACS blots and (**b**) column scatter graphs of WT versus RAGE−/− animals for Fas^hi^GL7^hi^ GC B cells, CXCR5^hi^PD1^hi^ TFH cells, LC^+^CD138^hi^ plasmacells/-blasts and CD21^lo^CD23^hi^ B220^+^ follicular B cells as well as CD21^hi^CD23^lo^ marginal zone B cells (FACS blot only). Each data point represents a single mouse, values are indicated as mean ± SD. Unpaired Mann–Whitney *U*-test was used for statistical analysis to determine differences between WT and RAGE−/− animals.

To examine if a RAGE deficiency affects the cellular immune phenotype, spleen were collected five months (replicate 1: *n* = 6 RAGE−/− and 7 WT mice) or seven months (replicate 3: *n* = 15 RAGE−/− and 10 WT mice) after pristane injection and flow cytometric analysis performed for the distribution of the following splenic cell subsets: plasmacells/-blasts (% CD138^hi^LC^+^/lymphocytes), B cells (% B220^+^/lymphocytes), mature B cells (% IgM^+^IgD^+^/B220^+^ B cells), follicular B cells (% CD21^lo^CD23^hi^/B220^+^ B cells), marginal zone B cells (% CD21^hi^CD23^lo^/B220^+^ B cells), B1 cells (% CD5^+^IgM^+^/B220^+^ B cells), germinal center B cells (% Fas^hi^GL7^hi^/B220^+^ B cells), CD4^+^ T cells (% CD4^+^/lymphocytes) and activated T cells (% CD69^+^ or % CD44^hi^/CD4^+^ T cells), Th1 and TH17 cells (% IFNγ^+^ and IL17^+^/CD4^+^ T cells), regulatory T cells (% FoxP3^+^/CD4^+^ T cells), follicular B helper T cells (TFH) (% CXCR5^hi^PD1^hi^/B220^-^CD4^+^ T cells), classic dendritic cells (% CD11c^hi^/leukocytes), neutrophils (Ly6G^hi^CD11b^+^/leukocytes) and inflammatory Ly6C^hi^ and resident Ly6C^lo^ monocytes/macrophages (Ly6C^hi^ and Ly6C^lo^CD11b^+^/leukocytes) (Table 1, Figure 2, Supplement Figure S3, Supplement Table S1). While T cell differentiation and activation, as well as distribution of innate immune cells, were similar in both groups (Table 1, Supplement Table S1), slight changes were detectable within the B cell compartment between RAGE−/− and WT animals (Table 1, Figure 2, Supplement Figure S3, Supplement Table S1). Interestingly, we observed a decrease in plasmacells/-blasts that are importantly involved in the production of anti-dsDNA antibodies. In one of two independent experiments, we further detected a decrease in germinal center B cells and increase in follicular B cells in RAGE−/− animals (Table 1, Figure 2, Supplement Figure S3, Supplement Table S1). GCs are structures within lymphoid tissue wherein B cells undergo clonal expansion, class-switch recombination, and affinity maturation. The fact that many pathologic autoantibodies are high-affinity class-switched IgG antibodies suggests an involvement of the GC reaction in their pathogenesis [34]. In one of the two independent experiments we also noted a slight, but not significant decrease of TFH cells in RAGE−/− animals (Figure 2, Supplement Figure S3).

To summarize, we observed a tendentious but not stringent reduction in GC B cells and antibody-secreting plasmacells/-blasts that is consistent with the slightly reduced anti-dsDNA autoantibody titers (Figure 1b, Supplement Figure S1a,b) in RAGE−/− animals.

### 2.3. Influence of RAGE Deficiency on Development of Arthritis

**Figure 3:**
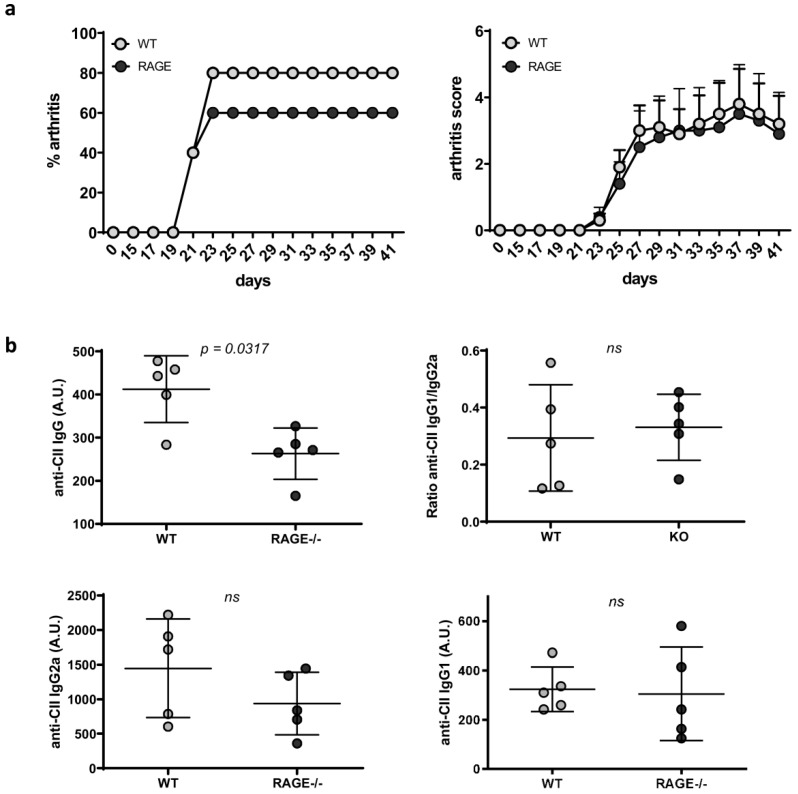
Influence of RAGE deficiency on autoantibody levels and disease development in CIA arthritis. (**a**) Mean arthritis scores and cumulative disease incidence in cCII-immunized RAGE−/− versus WT mice. Each data point represents the mean + SEM per group and time point (replicate 1: *n* = 5 RAGE−/− and 5 WT mice). (**b**) Serum concentrations of anti-CII IgG, anti-CII IgG2a and anti-CII IgG1 were analyzed in WT and RAGE−/− mice at day 41 after cCII-immunization. Each data point represents a single mouse, values are indicated as mean ± SD (replicate 1: *n* = 5 RAGE−/− and 5 WT mice). Unpaired Mann–Whitney *U*-test was used for statistical analysis.

Although RAGE-deficiency went along with some, if only slight immunologic changes, it did not significantly contribute to the development of lupus. As RAGE deficiency appeared to particularly affect B cell differentiation and associated autoantibody production, we decided to test its importance in an additional model of antibody-mediated autoimmunity, collagen-induced arthritis (CIA). In this model, genetically susceptible mice are immunized with a type II bovine collagen emulsion leading to auto-antibody production and disease development at roughly 21 days after immunization. In accordance with the current literature, the standard CIA immunization protocol hardly induced arthritis in animals on a C57BL/6 (H-2b) background (data not shown). We, therefore, employed an adopted protocol [35] and injected WT and RAGE−/− animals with a type II chicken collagen emulsion. In both, WT and RAGE−/− mice, arthritis could be induced in up to 60–80% of immunized animals (Figure 3a, Supplement Figure S4a). However, RAGE deficiency did neither affect disease penetrance, nor disease severity scores (Figure 3a, Supplement Figure S4a). In addition to the evaluation of clinical disease symptoms, we also examined anti-CII autoantibody titers. Alike autoantibody levels in pristane-induced lupus (Figure 1b, Supplement Figure S1a,b), in one of two independent experiments we observed an increase in anti-CII IgG in WT compared to RAGE−/− mice, while anti-CII IgG2a and anti-CII IgG1 subclasses, as well as the anti-CII IgG1/IgG2a ratio, did not differ between the groups (Figure 3b, Supplement Figure S4b). 

**Figure 4:**
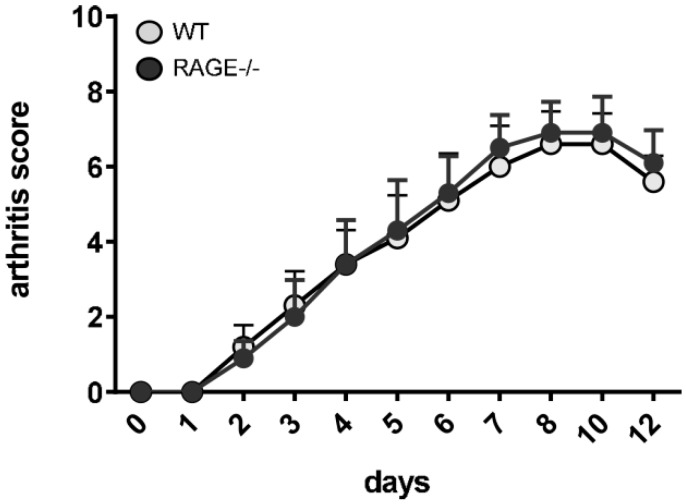
Influence of RAGE deficiency on disease development in K/BxN serum-transfer arthritis. Mean arthritis scores of RAGE−/− versus WT mice (replicate 1: *n* = 5 mice per group) after intraperitoneal injection of arthritogenic K/BxN serum. Values are indicated as mean + SEM. Unpaired Mann–Whitney *U*-test was using for statistical analysis.

In accordance with our results in pristane-induced lupus, these results point towards a slight, but not stringent effect of a RAGE-deficiency on autoantibody production, while disease severity was not affected (Figure 1, Supplement Figures S1 and S2). This may indicate that RAGE rather affects the adaptive immune than innate effector phase. To dissect RAGE effects on the early adaptive versus late innate disease phase, we additionally employed the K/BxN serum transfer model. While pristane-induced lupus as well as CIA cover both phases of autoimmune pathogenesis, early immune phase, mainly driven by adaptive immunity, as well as innate effector phase, mainly driven by an infiltration of macrophages and neutrophils, the injection of arthritogenic serum and resulting immune complex formation circumvents the adaptive immune phase and elicits arthritis symptoms within days [36]. In both RAGE and WT animals, treatment with K/BxN serum induced highly active arthritis with no difference in disease severity (Figure 4, Supplement Figure S5), arguing against an impact of RAGE-signaling on the effector phase of joint inflammation, which is predominantly driven by innate immune cells. In support of that fact, we did not notice changes in immune cell infiltration in kidneys of WT and RAGE−/− animals five or seven months after pristane injection (data not shown). 

To summarize, in both, pristane-induced lupus and CIA, RAGE deficiency causes only a slight reduction in autoantibody concentrations but has no effects on nephritis development or joint inflammation. 

## 3. Discussion

RA and SLE are characterized by the appearance of antibodies directed to self-antigens and the formation of inflammation-inciting immune-complexes. Here, it has become increasingly clear that PRR activation through DAMPs and PAMPs may importantly contribute to the break of tolerance that underlies autoimmune pathogenesis by helping to render seemingly irrelevant antigens more immunogenic [37]. In this study, we have examined the relevance of the PRR RAGE in antibody-mediated autoimmunity employing RAGE−/− animals in murine models of RA and lupus. Generally, in both pristane-induced lupus and CIA arthritis, RAGE deficiency caused only a slight reduction in autoantibody levels that was paralleled by a tendentious, but not consistent decrease in plasma cells, germinal center B cells and increase in follicular B cells. These changes may, however, not be stringent enough to inflict significant effects on nephritis development or joint inflammation. 

Rather than playing a key role in the break of immunological tolerance, we assume that RAGE-signaling may collaborate with or assist other receptors linking environmental factors, PAMPs or DAMPs, to trigger autoreactive immune responses in RA and SLE. For instance, such mediator/collaborator function may lie in the capacity of RAGE to detect nucleic acids in vivo. By promoting DNA uptake into endosomes, RAGE is able to control access of nucleic acids from the extracellular space towards secondary receptors, such as TLR7 and TLR9, able to trigger downstream pro-inflammatory cascades [38]. However, apart from RAGE, further cell surface receptors, able to bind and internalize nucleic acids, have been discovered, such as DEC-205 [39], CD11b/CD18 [40] or class A scavenger receptors [41], suggesting redundancy. 

Apart from mediating ligand internalization, RAGE can also directly activate pro-inflammatory pathways upon binding to ligands, such as HMGB1. Although RAGE is believed as HMGB1’s preferred receptor, the pro-inflammatory effects of HMGB1 may not necessarily depend on RAGE only. HMGB1 and AGEs also engage further PRRs, such as TLR2 and TLR4 [42]. Like RAGE, TLR2 and TLR4 can contribute to the establishment of an inflammatory response and disease pathogenesis through NF-κB activation and the release of pro-inflammatory cytokines [43].

Studies from our laboratory proved that in pristane-induced lupus, TLR2 is required for autoantibody production and development of renal disease [44]. In this model, TLR2 deficiency alone reduced the number of splenic plasma cells, release of IL6 and IL10 and recruitment of dendritic cells. Moreover, in TLR2- and TLR4-deficient C57BL/6lpr/lpr, as well as pristane-injected TLR4-deficient animals, development of murine lupus, was mitigated, while TLR2 and TLR4 activation enhanced disease [45,46,47,48]. In murine arthritis models, deficiencies in TLR4 and TLR2 inflicted divergent results, with TLR2 showing disease-mitigating effects, while TLR4 stimulation triggered arthritis [48,49,50]. TLR2 and TLR4 were also examined in SLE and RA patient samples and were shown to be differently expressed [48,51,52,53,54] compared to healthy controls. Moreover, cells from patients displayed dysregulated responsiveness towards TLR4 and TLR2 ligation regarding the secretion of inflammatory cytokines and molecules relevant to disease pathogenesis [48,55,56,57].

Like TLR2 and TLR4, also RAGE expression increases in diverse inflammatory and stress conditions, which suggests a possible contribution to SLE and RA pathogenesis [22,58,59]. In support of that, also AGEs and HMGB1 accumulated and correlated positively with disease course and manifestation [58,60,61,62,63], while circulating sRAGE concentrations were found to be decreased in patients with RA [64], SLE [65] and juvenile idiopathic arthritis [66]. Moreover, RAGE polymorphisms were shown to be associated with susceptibility and disease in lupus nephritis [67].

While these studies consistently point towards a disease triggering role of RAGE, functional murine studies on the relevance of RAGE in lupus, including ours, show rather divergent results. While in our studies RAGE deficiency did not significantly impact disease initiation and severity, treatment with sRAGE improved nephritis in lupus-prone NZB/W F1 animals [68]. In contrast to that, RAGE deletion in B6-MRL.Fas.lpr/j mice exacerbated lymphoproliferation as a consequence of reduced caspase 3 activation and delayed apoptosis in splenic T cells, exacerbating the lupus phenotype [69].

There are different explanations for the observed discrepancies. First, while in our study the effects of a selective RAGE deficiency were examined, application of the soluble decoy receptor sRAGE will most probably yield much broader effects as sRAGE simultaneously prevents the engagement of multiple PRRs by soluble ligands, such as HMGB1. Moreover, RAGE antagonism may have off-target effects on molecules that have not yet been identified as RAGE ligands [69]. 

Second, it needs to be taken into consideration that different models of murine lupus have been employed. Although lupus in pristane-induced and B6-MRL.Fas.lpr/j mice is characterized by the production of anti-dsDNA autoantibodies, lymphoproliferation and IC-mediated glomerulonephritis, distinct molecular and immunologic mechanisms underlie these pathologies. B6-MRL.Fas.lpr/j mice carry a loss of function mutation in the death receptor Fas/CD95 that inflicts massive lymphoproliferation as a result of defective apoptosis [69,70]. In humans, this defect is known as autoimmune lymphoproliferative syndrome (ALPS). ALPS shares many symptoms with SLE although glomerulonephritis is uncommon in ALPS patients [33,71]. Contrary to the defined genetic defect in B6-MRL.Fas.lpr/j mice and the associated phenotype that is mostly related to the lymphoproliferation (lpr) gene, in most cases, SLE is a genetically more complex disease, in which a combination of several allelic susceptibility variants is believed to lead to epistatic interactions that enhance the overall contribution by each locus [1,33,69]. As discussed by Goury et al. [69], the aggravated phenotype induced by RAGE deletion in the MRL/lpr genetic background may result from specific features of this lupus model and might not reflect the effect of RAGE blockade in lupus. 

Further, in this study, the employed model of pristane-induced lupus may have weaknesses that need to be considered. Though it allowed examining effects of a selective RAGE deficiency, disease symptoms and autoantibody levels are rather weak compared to other models, such as MRL/lpr or NZB/W F1 mice. This can make it difficult to estimate clear differences between WT and KO mice. However, rather weak models like pristane-induced lupus may have the advantage to show even slight differences induced by a genetic modification. Alternative approaches may involve the use of stronger spontaneous lupus models, such NZB/W F1 animals and the use of RAGE pharmacological inhibitors that could be applied at different disease stages. As RAGE is pursued as an attractive therapeutic target, several strategies have been addressed to inhibit RAGE signaling [72]. Being also of relevance in the clinical setting, RAGE inhibitors should be examined in future experiments and different autoimmune settings. Likewise, it would be of interest to evaluate the effects of treatment of RAGE−/− and WT animals with sRAGE in autoimmune diseases. The application of sRAGE acting as a decoy receptor for RAGE ligands may exert beneficial effects by preventing proinflammatory signaling not only via RAGE but also via other PRR. RAGE−/− mice also lack sRAGE, hence signaling through other PRR, such as TLR2 and TLR4, might be even increased and may partially compensate for the lack of proinflammatory signaling via RAGE.

Regarding autoimmune arthritis, to the best of our knowledge, this is the first in vivo study to directly examine the selective effect of RAGE signaling. Like in pristane-induced lupus, we detected a partial reduction in autoantibody production, however, no difference in arthritis severity in both CIA and K/BxN serum transfer models, with the latter selectively covering the effector phase of joint inflammation. The influence of RAGE-signaling on joint inflammation has also been addressed in models of non-autoimmune arthritis that show divergent results. For instance, after intra-articular injection of *S. aureus*, RAGE deficiency significantly attenuated the frequency and severity of septic arthritis in one study [73]. However, RAGE deficiency did not affect disease in another study [74] and did also not affect osteoclast differentiation and function in antigen-induced arthritis (AIA) [75]. Nevertheless, most studies point towards the anti-inflammatory effects of sRAGE. In arthritic IL1Ra−/− mice, overexpression of sRAGE ameliorated arthritis through decreasing TH17 and reciprocally increasing Treg. In CIA, the use of a bone-targeting therapeutic form of sRAGE significantly ameliorated inflammatory arthritis, while untagged sRAGE showed little effectiveness [76].

Although not fully consistent, in most circumstances and autoimmune diseases, including diabetes, cardiovascular disease and nephropathy [9,16,17,18,22,23,29,58], increased RAGE signaling may go along with pro-inflammatory effects. The relevance of signaling via RAGE versus other PRR may vary depending on disease, antigenic structure, receptor expression, and predominantly contributing (immune) cells. Moreover, redundancy and interplay between different signaling pathways may prevail, and further studies are needed to dissect these cross-talks [77]. On an immunological level, effects of RAGE-signaling appear to be versatile. For instance, RAGE was shown to be critically involved in pDC and B cell activation in concert with TLR9, it affected DC maturation, migration, and interaction with NK cells as well as B and T cell activation and differentiation [58]. In our study, a RAGE-deficiency had only slight effects on the immune status and mainly provoked changes in the B cell compartment and autoantibody production. 

To conclude, the observed slight effects on immune cells and autoantibodies with no significant influence on disease severity argue against the fact that RAGE plays a major role in linking the appearance of environmental factors to the initiation and propagation of antibody-mediated autoimmunity. Other PRR, such as TLR2 and TLR4, may have a greater impact on these diseases. Moreover, the contribution and interplay with further AGEs and associated signaling cascades may play a role, which needs to be addressed in future studies.

## 4. Materials and Methods

### 4.1. Animals and Treatments

Animal experiments were approved by the local governmental commission for animal protection of Freiburg (Regierungspräsidium Freiburg, approval no. G16/56 and G19/23). C57BL/6 and RAGE−/− animals (C57BL/6 background) were housed in the animal facilities of the University of Erlangen–Nuremberg and CEMT in Freiburg. RAGE−/− animals were kindly provided by Prof. Angelika Bierhaus and Prof. Peter Nawroth (University of Heidelberg, Germany). Absence of RAGE in the employed RAGE−/− mice has been confirmed by previous studies [78,79]. All mice were maintained under conventional housing with five mice per cage. The mice were maintained under controlled 12 h light/ 12 h dark cycles. For induction of pristane-induced lupus, eight weeks old animals were injected intraperitoneally with a single 500 μL dose of pristane (Sigma-Aldrich/Merck, Darmstadt, Germany). Pristane-treated animals were killed at five months (independent replicate 1: *n* = 6 RAGE−/− and 7 WT mice), six months (independent replicate 2: *n* = 6 RAGE−/− and 8 WT mice) or seven months (independent replicate 3: *n* = 15 RAGE−/− and 10 WT mice) after pristane injection. Animals were saline-perfused and spleens, sera, kidneys, urine, and intraperitoneal lipogranulomas were collected to determine anti-dsDNA autoantibody titers (ELISA), proteinuria (BCA), nephritis severity (histology), immune status (FACS), spleen and lipogranuloma weights. Immune status and kidney histology were only examined in replicate 1 and 3. For induction of CIA, eight-week-old animals were intradermally injected with a 100 μL emulsion containing CFA supplemented with 4mg/mL mycobacterium tuberculosis and 2 mg/mL chicken collagen II (cCII) at equal volumes. CIA induction on an H-2^b^ background requires higher concentrations of CFA for sufficient activation of dendritic cells and priming type II collagen-reactive T cells in draining lymph nodes [80]. Two independent experiments were performed (replicate 1: *n* = 5 RAGE−/− and 5 WT mice; replicate 2: *n* = 8 RAGE−/− and 7 WT mice). Arthritis scores were determined every two days. All animals were sacrificed at day 41 post immunization for serum collection and determination of anti-CII Ig. For induction of serum-transfer arthritis, 180 µL K/BxN serum was injected intraperitoneally. Two independent experiments were performed (replicate 1: *n* = 5 RAGE−/− and 5 WT mice; replicate 2: *n* = 7 RAGE−/− and 7 WT mice) and arthritis scores determined every one to two days.

### 4.2. Arthritis Scoring

The clinical severity of arthritis was scored as previously described [34,81,82]. All four paws were scored based on a scale of 0 to 3, and severity was described as the cumulative score.

### 4.3. Assessment of Kidney Disease 

To assess urinary protein excretion, the mice were placed in metabolic cages (Tecniplast, Hohenpeißenberg, Germany), and urine samples were collected over a 24-hour period. Protein concentrations were determined using a BCA protein assay (Pierce/Thermo Fisher Scientific, Freiburg im Breisgau, Germany) according to the manufacturer’s instructions.

Kidneys were sampled from saline-perfused RAGE−/− and age-matched C57BL/6 control mice, fixed in 4% paraformaldehyde buffered in PBS pH 7.4 and embedded in paraffin. Two-µm-thick paraffin sections were cut and stained with periodic acid Schiff (PAS) using standard protocol. Renal morphology was investigated by light microscopy at 400x magnification. Glomeruli were scored for lupus-induced changes including mesangial matrix expansion and focal glomerulosclerosis and graded using a semi-quantitative score ranging from 0–4: 0 = healthy glomeruli without pathological changes, 1 = up to 25% (low), 2 = 25–50% (moderate), 3 = 50–75% (marked) and 4 = 75% (very marked) of the glomerular cross-section showed mesangial matrix accumulation and focal sclerosis. Glomerular changes were shown as mean score of 30 glomeruli.

### 4.4. Determination of Serum Autoantibodies 

#### 4.4.1. Anti-CII IgG Antibodies and Its Subclasses

Serum levels of anti-CII IgG, anti-CII IgG2a and anti-CII IgG1 were measured by ELISA. Microtiter plates were coated with 10 μg/mL native chicken CII, blocked with 2% BSA/PBS and then incubated with mouse sera diluted in PBS. Bound IgG and IgG subclasses were detected by incubation with HRP-conjugated rabbit anti-mouse IgG, respectively, with anti-mouse IgG2a and anti-mouse IgG1. For color development, TMB substrate (Thermo Fisher Scientific, Freiburg im Breisgau, Germany) was used according to the manufacturer’s instructions and the optical density (OD) read at 450 nm with an ELISA Reader Infinite F50 (Tecan, Crailsheim, Germany) with a reference measurement at 620 nm. 

#### 4.4.2. Anti-dsDNA Autoantibodies

IgG antibody secretion to dsDNA was determined by enzyme-linked immunosorbent assay (ELISA) as previously described [44]. Briefly, microtiter plates (NUNC, MaxiSorp, 96 well) were coated with 20 µg/mL poly–l-lysine (Sigma/Merck, Darmstadt, Germany) in TE buffer for 1 h at 37 °C and, subsequently, with 20 µg/mL calf thymus DNA (Sigma) in TE buffer overnight and then blocked with 2% fetal calf serum (FCS) in PBS. Thereafter, serum samples diluted in 2% FCS in PBS were added and incubated for 2 h at room temperature. We detected bound anti-dsDNA IgG by incubation with goat anti-mouse IgG Fc-γ HRP (Jackson ImmunoResearch/Dianova, Hamburg, Germany) followed by development with TMB substrate (Thermo Fisher Scientific, Freiburg im Breisgau, Germany) according to the manufacturer’s instructions. The absorbance was measured using the TECAN Photometer infinite F50 using a wavelength of 450 nm with a reference measurement at 620 nm. To achieve comparability between the different plates and assays, diluted sera from nephritic reference animals with high and low anti-dsDNA titers (S1 and S2) were added to each ELISA plate and the auto-antibody titer expressed as arbitrary unit in relation to the difference between high and low reference serum (S1–S2). 

### 4.5. Flow Cytometry

Flow cytometric analysis was performed on spleen single cell suspensions using fluorochrome-conjugated monoclonal antibodies. For intracellular cytokine staining, splenocytes were restimulated for four hours with 50 ng/mL Phorbol-12-myristat-13-acetat (PMA) and 1µg/mL ionomycin (Sigma-Aldrich) in the presence of brefeldin A (ebiosciences), thereafter fixed and permeabilized using the BD Cytofix/Cytoperm Kit according to the manufacturer’s instructions. For intranuclear staining, splenocytes were fixed and permeabilized using the eBioscience FoxP3/Transcription Factor Staining Buffer Set according to the manufacturer’s instructions. The following antibodies were used: APC-conjugated anti-CD11c (eBioscience/ Thermo Fisher Scientific, Freiburg im Breisgau, Germany), PE-conjugated anti-CD11b (BioLegend, Koblenz, Germany), V450-labelled anti-Ly6G (BD), PE-Cy7-conjugated anti-Ly6C (BD), APC-Cy7-conjugated anti-B220 (BioLegend), PerCP-conjugated anti-CD8 (BioLegend), Pacific Blue-conjugated anti-CD4 (BioLegend), PE-Cy7-conjugated anti-CD4 (BioLegend), APC-conjugated anti-FoxP3 (eBioscience), PE-conjugated anti-CD138 (BD), Biotin-conjugated anti-λ1, λ2, and λ3 Light Chain (BD), Biotin-conjugated anti-κ Light Chain (BD), V450-conjugated Streptavidin (BD), APC-conjugated anti-CD21/CD35 (BioLegend), PE-conjugated anti-CD23 (BD), PE-Cy7-conjugated anti-IgM (BioLegend), PE-conjugated anti-CD5 (Biolegend), Alexa-Fluor 647-conjugated anti-IgD (BioLegend), PE-conjugated anti-IL17 (eBioscience), APC-conjugated anti-IFNγ (BioLegend), PerCP-Cyanine 5.5-conjugated Streptavidin (eBioscience), PE-conjugated anti-PD1 (eBioscience), APC-conjugated anti-CD44 (BD), V450-conjugated anti-CD69 (BD), Pacific Blue-conjugated anti-GL7 Antigen (BioLegend), PE-conjugated anti-mouse CD95 (BD). Prior to staining, cells were Fc-blocked with rat anti-mouse CD16/32 (Biolegend). All surface staining steps were performed for 30 min on ice in FACS buffer (2 % FCS in PBS). 

### 4.6. Statistical Analysis

*p* Values were calculated by unpaired Mann–Whitney *U* test, using Instat software (GraphPad). *p* Values less than or equal to 0.05 were considered significant.

## Figures and Tables

**Table 1:** Frequency of splenic cell subsets in WT and RAGE−/− animals.

Splenic Cell Subsets		WT	RAGE−/−
CD11c^hi^	/	1.448 (0.226)	1.208 (0.222)
CD11b^+^	/	6.369 (1.127)	5.973 (1.697)
	Ly6G^hi^	3.351 (0.838)	2.736 (1.199)
	Ly6C^hi^	0.918 (0.376)	1.090 (0.356)
	Ly6C^lo^	1.917 (0.454)	1.980 (0.387)
CD4^+^	/	13.740 (1.225)	14.686 (2.554)
	CD44^hi^	54.810 (4.043)	54.486 (7.926)
	CD69^+^	24.690 (2.591)	26.766 (5.135)
	CXCR5^hi^PD1^hi^	11.119 (2.381)	10.789 (2.735)
	IFNγ	10.666 (3.198)	10.064 (1.686)
	IL17	0.365 (0.232)	0.403 (0.134)
	FoxP3	26.944 (6.147)	27.206 (4.679)
B220^+^	/	50.620 (3.572)	57.600 (4.227)
	Fas^hi^GL7^hi^ (*)	6.554 (4.515)	4.515 (1.618)
	CD21^lo^CD23^hi^ (**)	45.530 (8.893)	52.186 (6.125)
	CD21^hi^CD23^lo^	6.360 (3.437)	6.892 (2.670)
	IgD^+^IgM^+^	24.550 (7.334)	27.773 (6.501)
	IgM^+^CD5^+^	1.117 (0.288)	1.106 (0.249)
	LC^+^CD138^hi^ (***)	1.785 (0.324)	1.453 (0.397)

The analysis was performed by flow cytometry seven months after pristane injection. Values are the mean +/− SD (replicate 3: *n* = 15 RAGE−/− and 10 WT mice). Unpaired Mann–Whitney *U*-test was used for statistical analysis to determine differences between WT and RAGE−/− animals. * p = 0.0009, ** p = 0.0475, *** p = 0.0254.

## Supplementary Materials

Supplementary materials can be found at https://www.mdpi.com/1422-0067/20/13/3234/s1.

## Abbreviations

RAGE	receptor for advanced glycation end products
CIA	collagen-induced
ELISA	enzyme-linked immunosorbent assay
SLE	systemic lupus erythematosus
RA	rheumatoid arthritis
dsDNA	double-stranded DNA
PAMPs	pathogen-associated molecular patterns
DAMPs	danger-associated molecular patterns
PRRs	pattern recognition receptors
DCs	dendritic cells
AGEs	advanced glycation end products
HMGB1	high mobility group box 1
sRAGE	soluble receptor for advanced glycation end products
ALPS	autoimmune lymphoproliferative syndrome
Lpr	lymphoproliferation
PAS	periodic acid Schiff
OD	optical density
FCS	fetal calf serum
A.U.	arbitrary unit
