# Supplementary material for: Relevance of Receptor for Advanced Glycation end Products (RAGE) in Murine Antibody-Mediated Autoimmune Diseases
Source: Int J Mol Sci. 2019 Jul 1;20(13):3234. doi: 10.3390/ijms20133234 (PMC6651235; doi:10.3390/ijms20133234)
Supplement: Supplementary file 1 [file ijms-20-03234-s001.pdf]

Supplement Figure 1

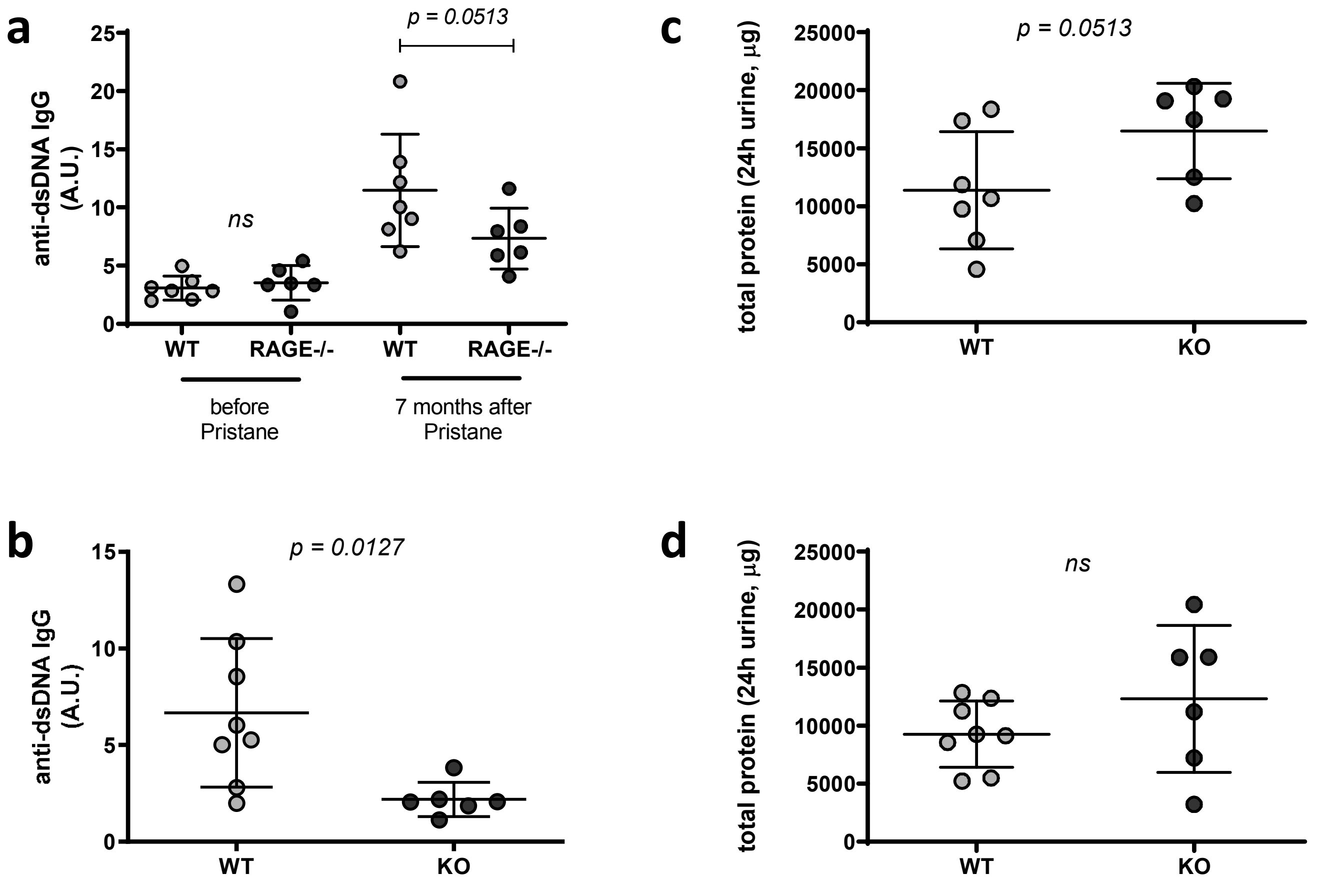

Supplement Figure 1. Influence of RAGE deficiency on autoantibody levels and proteinuria in pristane-induced lupus. C57BL/6 (WT) and RAGE-/- mice were injected intraperitoneally with 0.5ml pristane. Serum and urine was collected five months (replicate 1: n = 6 RAGE-/- and 7 WT mice) **(a + c)** or six months (replicate 2: n = 6 RAGE-/- and 8 WT mice) **(b + d)** later. The concentrations of anti-dsDNA autoantibodies were determined by ELISA (left). Protein concentrations were determined using a BCA protein assay in urine samples collected for 24 hours using metabolic cages (right). Each data point represents a single mouse; values are indicated as mean ± SD. Unpaired Mann-Whitney *U*-test was used for statistical analysis. Note that for replicate 1, anti-dsDNA autoantibody levels were compared before and five months after pristane injection in C57BL/6 (WT) and RAGE-/- animals. After five months, anti-dsDNA autoantibodies were ca. 2.3 fold higher in RAGE-/- and ca. 3.7 fold higher in WT animals **(a)**.

Supplement Figure 2

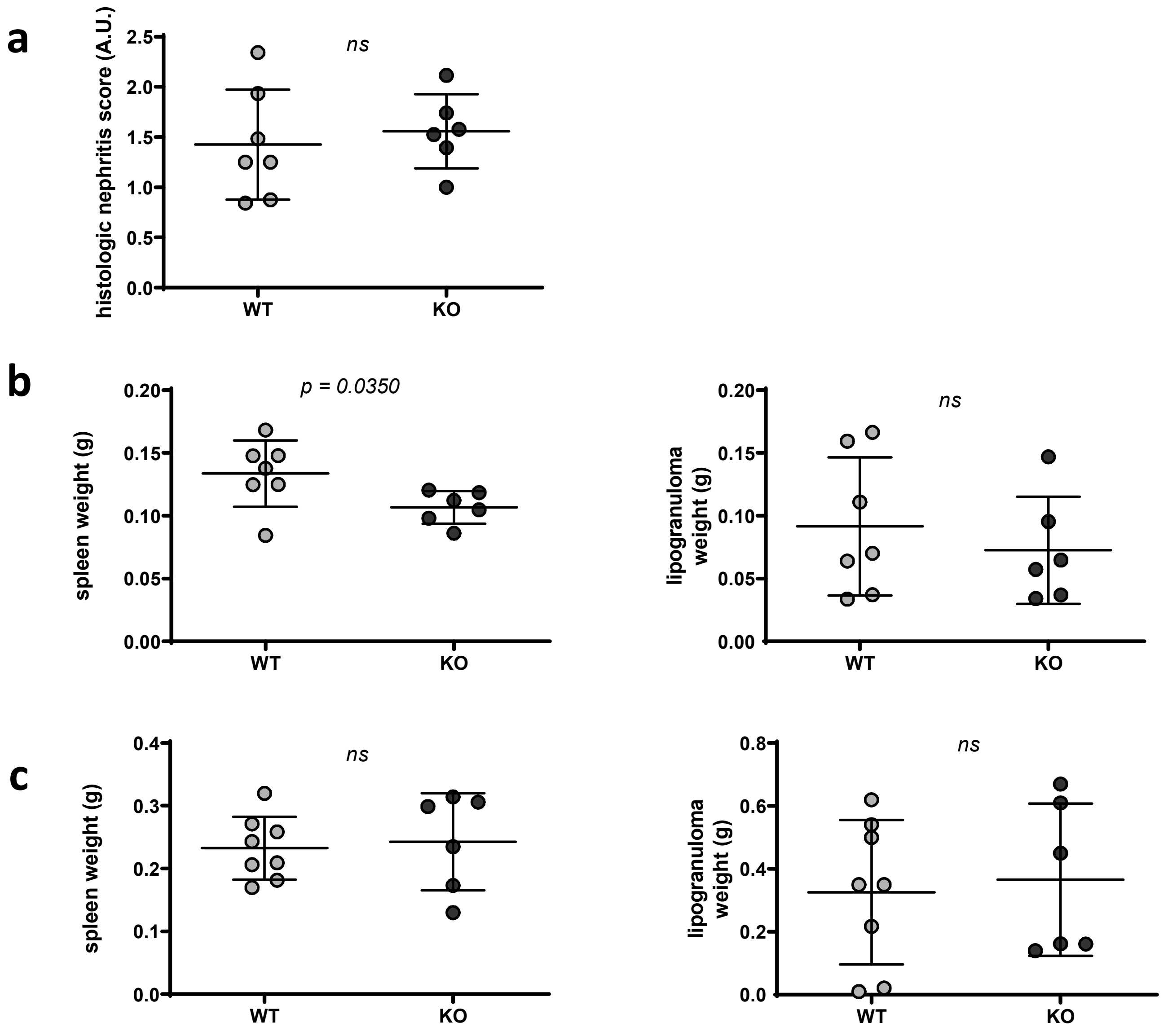

Supplement Figure 2. Influence of RAGE deficiency on spleen and lipogranuloma weights as well as histologic nephritis score. C57BL/6 (WT) and RAGE-/- mice were injected intraperitoneally with 0.5ml pristane. Spleen and kidney samples as well as intraperitoneal lipogranulomas were collected five months (replicate 1: n = 6 RAGE-/- and 7 WT mice) **(a, b)** or six months (replicate 2: n = 6 RAGE-/- and 8 WT mice) **(c)** later. **a)** Determined were cumulative nephritis scores of RAGE-/- compared to WT animals. **b + c)** Determined were weights of spleen (left) and lipogranulomas (right). Each data point represents a single mouse; values are indicated as mean ± SD. Unpaired Mann-Whitney *U*-test was used for statistical analysis.

Supplement Figure 3

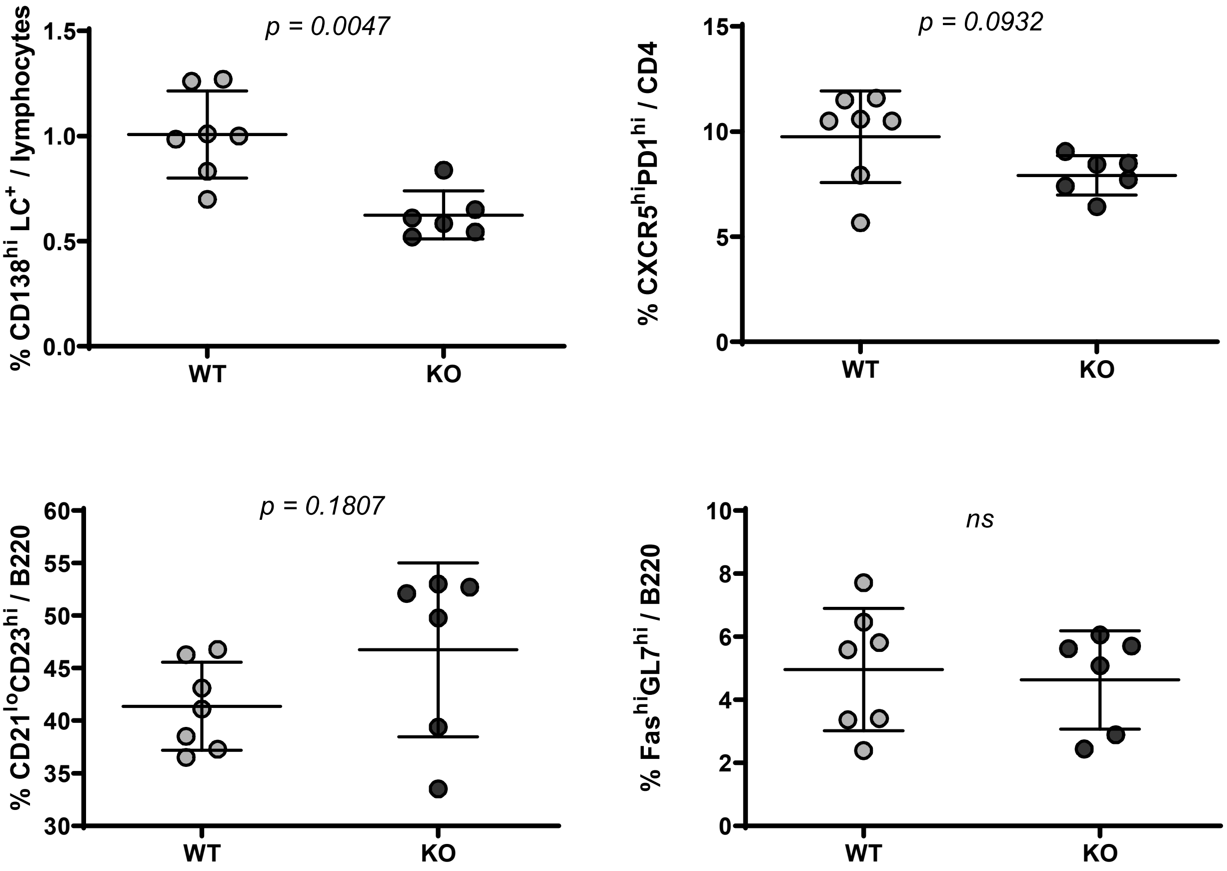

Supplement Figure 3. Phenotype of splenocytes in WT and RAGE-/- mice after pristane injection. The analysis was performed by flow cytometry 5 months after pristane injection (replicate 1: n = 6 RAGE-/- and 7 WT mice). Depicted are column scatter graphs of WT versus RAGE-/- animals for $Fas^{hi}GL7^{hi}$ GC B cells, $CXCR5^{hi}PD1^{hi}$ TFH cells, $LC^{+}CD138^{hi}$ plasmacells/-blasts and $CD21^{lo}CD23^{hi}$ $B220^{+}$ follicular B cells. Each data point represents a single mouse, values are indicated as mean ± SD. Unpaired Mann-Whitney *U*-test was used for statistical analysis to determine differences between WT and RAGE-/- animals.

Supplement Figure 4

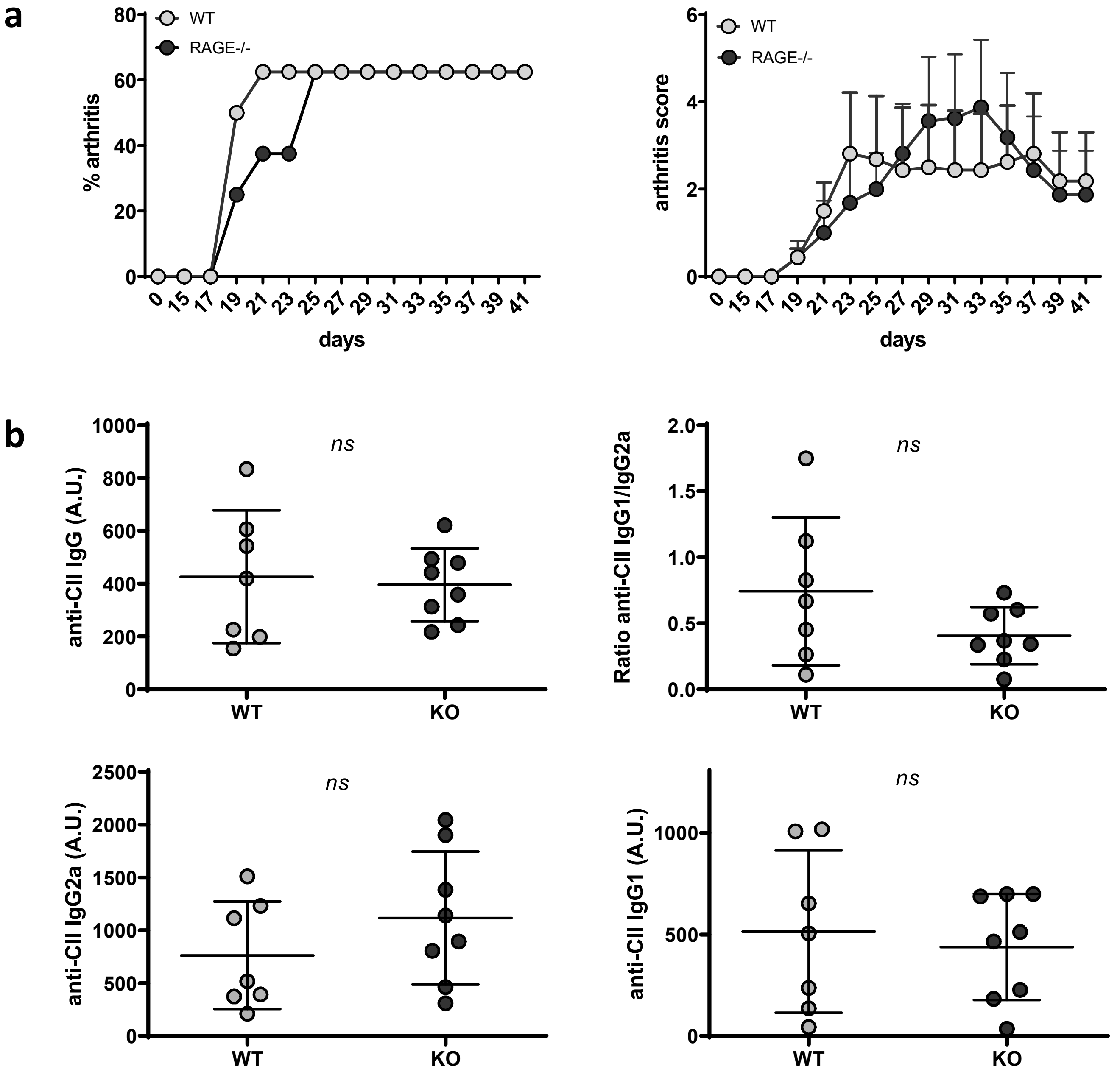

Supplement Figure 4. Influence of RAGE deficiency on autoantibody levels and disease development in CIA arthritis. (a) Mean arthritis scores and cumulative disease incidence in cCII-immunized RAGE-/- versus WT mice. Each data point represents the mean + SEM per group and time point (replicate 2: n = 8 RAGE-/- and 7 WT mice). **(b)** Serum concentrations of anti-CII IgG, anti-CII IgG2a and anti-CII IgG1 were analyzed in WT and RAGE-/- mice at day 41 after CII-immunization. Each data point represents a single mouse, values are indicated as mean ± SD (replicate 2: n = 8 RAGE-/- and 7 WT mice). Unpaired Mann-Whitney *U*-test was used for statistical analysis.

Supplement Figure 5

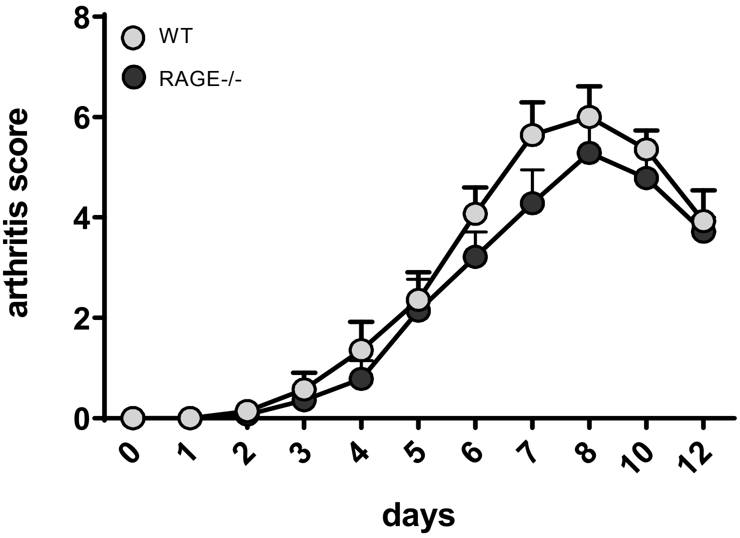

Supplement Figure 5. Influence of RAGE deficiency on disease development in K/BxN serum-transfer arthritis. Mean arthritis scores of RAGE-/- versus WT mice (replicate 2: n = 7 mice per group) after intraperitoneal injection of arthritogenic K/BxN serum. Values are indicated as mean + SEM. Unpaired Mann-Whitney *U*-test was used for statistical analysis.

Supplement Figure 6

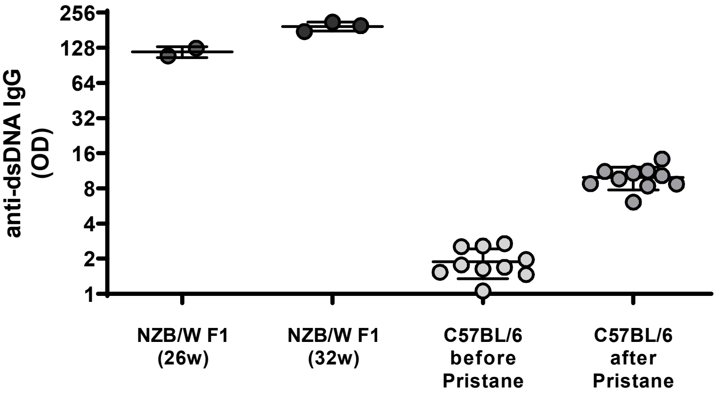

Supplement Figure 6. Anti-dsDNA antibody levels in pristane-induced lupus compared to NZB/W F1 mice. C57BL/6 (WT) mice were injected intraperitoneally with 0.5ml pristane. Serum was collected before as well as seven months after injection (n = 10 WT mice from replicate 3). Serum samples were also collected from 26w (n = 2) and 32w (n = 3) old NZB/W F1 animals. The concentrations of anti-dsDNA antibodies were determined by ELISA. Compared are OD values of 26w and 32w old NZB/W F1 animals to C57BL/6 animals before and 7 after pristane injection. Considering the dilution factor of each sample, the OD of anti-dsDNA autoantibodies from sera of 32w old NZB/W F1 mice was ca. 22 fold higher than that from pristane-treated animals, the OD from 26w old NZB/W F1 ca. 13 fold higher.

Supplement Figure 7

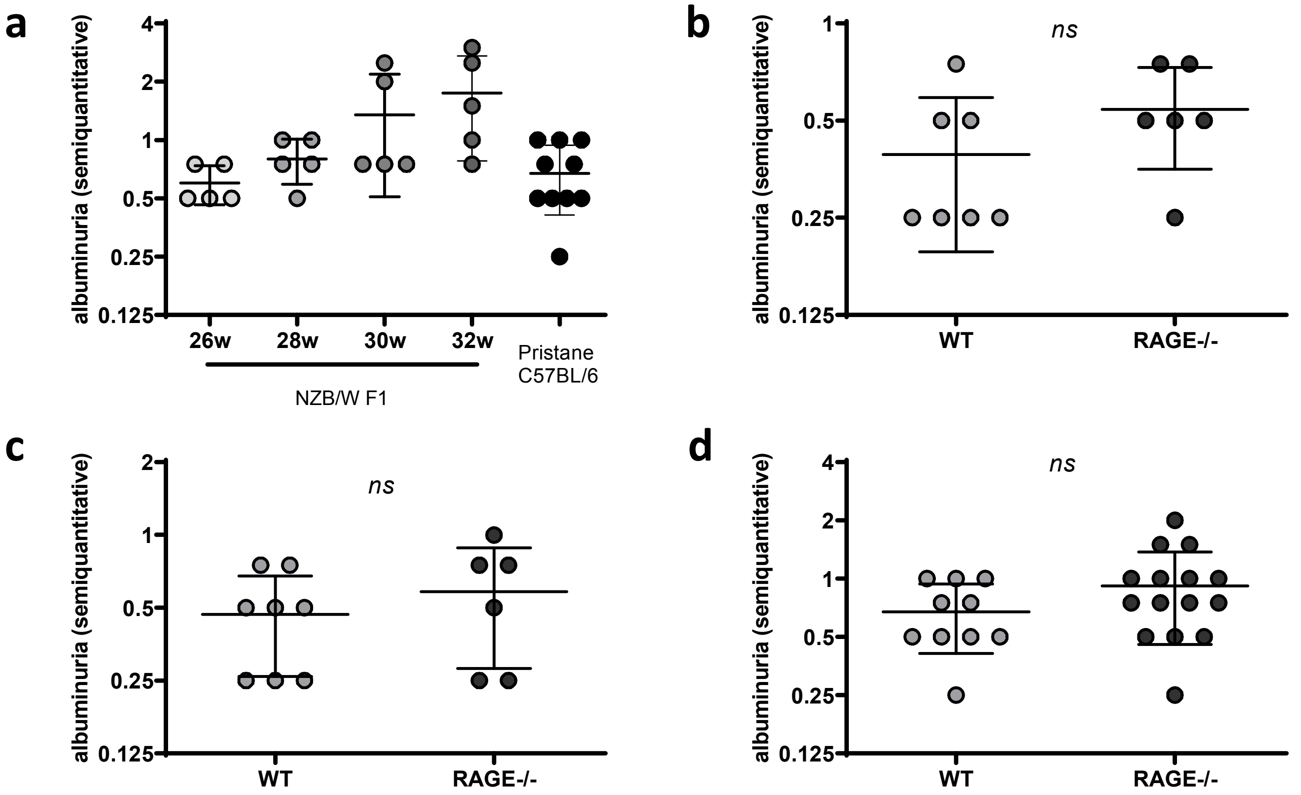

Supplement Figure 7: Semiquantitative albuminuria in pristane-induced lupus compared to NZB/W F1 mice. C57BL/6 (WT) and RAGE-/- mice were injected intraperitoneally with 0.5ml pristane. Urine was collected five months (replicate 1: n = 6 RAGE-/- and 7 WT mice), six months (replicate 2: n = 6 RAGE-/- and 8 WT mice) or seven months (replicate 3: n = 15 RAGE-/- and 10 WT mice) after injection. Urine samples were also collected from NZB/W F1 animals at 26w, 28w, 30w and 32w of age (n = 5). Albuminuria was determined semi-quantitatively using Albustix®. **a)** Compared are levels of albuminuria in NZB/W F1 animals at different ages to levels in C57BL/6 animals 7 months after pristane injection. **b – d)** compared are levels of albuminuria between RAGE-/- and WT animals 5 months **(b)**, 6 months **(c)** and 7 months **(d)** after pristane injection. Unpaired Mann-Whitney *U*-test was used for statistical analysis to determine differences between WT and RAGE-/- animals.

Supplement Table 1: Frequency of splenic cell subsets in WT and RAGE-/- animals

Splenic cell subsets		WT	KO
$CD11c^{hi}$		0.992 (0.201)	0.926 (0.137)
$CD11b^{+}$		6.551 (0.965)	5.553 (0.883)
	$Ly6G^{hi}$	2.648 (1.779)	1.88 (0.392)
	$Ly6C^{hi}$	1.3 (0.311)	0.998 (0.436)
	$Ly6C^{lo}$	2.765 (0.294)	2.429 (0.372)
$CD4^{+}$		15.714 (1.15)	15.392 (6.398)
	$CD44^{hi}$	50.285 (0.938)	46.2 (5.91)
	$CD69^{+}$	21.875 (2.96)	22.016 (2.153)
	$CXCR5^{hi}PD1^{hi}$	9.757 (2.171)	7.921 (0.938)
	IFNγ	7.39 (2.403)	7.256 (1.186)
	IL17	0.299 (0.043)	0.348 (0.062)
	FoxP3	29.785 (3.379)	26.75 (1.364)
$B220^{+}$		56.185 (5.67)	55.36 (2.37)
	$Fas^{hi}GL7^{hi}$	4.96 (1.937)	4.63 (1.559)
	$CD21^{lo}CD23^{hi}$	41.371 (4.1907)	46.75 (8.27)
	$CD21^{hi}CD23^{lo}$	4.295 (1.696)	4.496 (1.041)
	$IgD^{+}IgM^{+}$	8.882 (1.623)	10.073 (3.011)
	$IgM^{+}CD5^{+}$	0.473 (0.096)	0.394 (0.107)
	$LC^{+}CD138^{hi}$ (*)	1.01 (0.207)	0.624 (0.114)

The analysis was performed by flow cytometry five months after pristane injection. Values are the mean +/- SD (replicate 1: n = 6 RAGE-/- and 7 WT mice). Unpaired Mann–Whitney *U*-test was used for statistical analysis to determine differences between WT and RAGE−/− animals.* *p = 0.0047*
